# Influence of photoperiod on endogenous phytohormone levels and cadmium-related gene expression in *Sedum alfredii*

**DOI:** 10.1080/15592324.2025.2544317

**Published:** 2025-08-12

**Authors:** ShiMiao Chen, Bin Shan, Fuhai Zheng, Yanyan Li, QinYu Lu

**Affiliations:** a Key Laboratory, Guangxi Subtropical Crops Research Institute, Nanning, Guangxi, China; b Key Laboratory of Quality and Safety Control for Subtropical Fruit and Vegetable, Ministry of Agriculture and Rural Affairs, Nanning, China; c Guangxi Key Laboratory of Quality and Safety Control for Subtropical Fruits, Guangxi Subtropical Crops Research IInstitute, Nanning, Guangxi, China; d Agricultural Resources and Environmental Research Institute, Guangxi Academy of Agricultural Science, Nanning, Guangxi, China; e Fruit Tree Research Lab, Qinzhou Institute of Agricultural Sciences, Qinzhou, Guangxi, China

**Keywords:** Photoperiod, *Sedum alfredii*, phytohormones, cadmium, phytoremediation optimization

## Abstract

To determine whether photoperiod influences integrated physiological and molecular mechanisms underlying cadmium (Cd) uptake and tolerance in *Sedum alfredii*, plants were exposed to varying day lengths (2–24 h). Distinct photoperiod-dependent trends emerged: very short photoperiods primarily stimulated stress-related hormone signaling and early-stage flavonoid synthesis, while an intermediate photoperiod (~10 h) concurrently enhanced growth-promoting hormones, jasmonate signaling, and antioxidant defenses. This optimal photoperiod elicited a coordinated peak in phytohormonal responses, antioxidant enzyme activities, and Cd transporter gene expression. Canonical correspondence analysis identified three major co-regulatory modules integrating hormonal signaling, secondary metabolism, and heavy-metal transport. These modules included an auxin – IAA oxidase network, an ABA – phenolic metabolism axis linked to key metal transporter genes (*HMA* and *ZIP* families), and a cytokinin – flavonoid pathway associated with additional Cd transporters. The convergence of these modules underscores a systemic regulatory mechanism balancing plant growth, defense responses, and heavy-metal management. These findings provide a mechanistic understanding of how photoperiodic signals modulate endogenous hormone networks and associated molecular processes to optimize Cd accumulation and tolerance. This study thus identifies photoperiod as a pivotal environmental cue that could inform strategies to enhance *S. alfredii*‘s effectiveness in phytoremediation of Cd-contaminated soils.

## Introduction

Cadmium (Cd) pollution has become a major global environmental issue, posing significant risks to human health, agricultural productivity, and ecosystems.^1^ Cd contamination in soils, primarily from anthropogenic activities such as industrial discharge and mining, accumulates in crops like rice, vegetables, and wheat, entering the food chain and causing adverse health effects.^2^ Chronic exposure to Cd, even at low levels, can lead to serious health issues, including kidney damage, bone disorders, and an increased risk of cancer.^3^

To address this issue, researchers have explored several strategies. One approach involves applying biochar combined with iron and magnesium, effectively reducing Cd absorption in plants like spinach.^4^ Additionally, arbuscular mycorrhizal fungi have been shown to protect crops, mainly maize, from Cd toxicity.^5^ Furthermore, plant-based remediation techniques, such as using ryegrass enhanced with citric acid and Bacillus megaterium, have demonstrated promise in lowering Cd levels in contaminated soils,^6^ suggesting that phytoremediation is a relatively low-cost and effective solution for soil heavy metal pollution. Despite their ability to accumulate metals, hyperaccumulator plants face several challenges that limit their practical application. They often have low biomass and limited survivability in harsh environments, such as mine waste, which hinders their use in real-field settings.^7^ Additionally, not all hyperaccumulator plants grow rapidly enough to achieve significant metal uptake within a short period, which can delay remediation efforts.^8^ However, the most common issue is environmental adaptability. Some hyperaccumulator plants may not adapt well to varying soil conditions or ecological stresses, reducing their effectiveness at different contaminated sites.^9^

*Sedum alfredii*, a hyperaccumulator of Cd, Pb, and Zn, has a long history of being used to remediate soil contaminated by heavy metals. In long-term phytoremediation applications, hyperaccumulating plants’ heavy metal absorption ability tends to be unstable, varying with latitude, climate,^10^ agrotype,^11^ and pollution levels.^12^ The accumulation of seven heavy metals in the rhizosphere soil and three local pioneer plants (*Ageratum conyzoides*, *Conyza canadensis*, and *Miscanthus floridulus*) on barren land at the Mingshan coal mine tailings shows a clear dependence on geographic conditions.^13^ Additionally, *Amaranthus spp*. exhibited the highest cadmium accumulation in Huayuan, while lead accumulation was more significant in Yueyang and Liuyang across three Zn mining areas (Huayuan HYX, Yueyang LYX, and Liuyang LYX).^14^ This phenomenon is not limited to hyperaccumulators but is also observed in many crop species. In four common subtropical understory plants from southern China (*Euodia lepta*, *Ilex asprella*, *Mussaenda pubescens*, and *Rhodomyrtus tomentosa*), significant differences in Cu, Zn, Cd, and Pb concentrations were found across four sampling locations.^15^ Research on Oryza sativa has shown that temperature fluctuations can affect the uptake of micronutrients, including heavy metals.^16^ This instability poses challenges for the site-specific adaptation of hyperaccumulators in heavy metal pollution remediation and the cultivar selection of plants with low heavy metal accumulation. Therefore, understanding plants’ mechanisms and patterns of heavy metal uptake under various environmental conditions is crucial for controlling their absorption in agricultural production.

Photoperiod is a crucial factor when introducing species across regions. For many plants, photoperiod sensitivity (i.e., the plant’s response to changes in day length) significantly affects flowering time and adaptability, impacting growth and growth periods.^17^ In 18 ornamental sunflower varieties, 12 (66.7%) showed earlier bud formation under short-day conditions, and 16 (88.9%) exhibited a quantitative short-day response.^18^ Additionally, in natural field conditions across three latitudes in China, polymorphisms in the coding and non-coding regions of the *GmGBP1* gene were identified in 278 soybean germplasms, with five polymorphisms and four haplotypes in the gene promoter region linked to soybean flowering time and maturity.^19^ Under certain extreme conditions, photoperiods can inhibit flowering. For example, long-day photoperiods inhibit flowering in sunflowers,^20^ while strawberry plants do not form flower buds when day length exceeds a specific limit (usually > 12 h).^21^ Flowering marks a crucial transition in plants from vegetative to reproductive growth. Plants demonstrate distinct characteristics during the absorption of heavy metals at diverse growth stages. The accumulation of heavy metals in pea plants shows a remarkable growth-stage dependence, with the highest uptake rates from early flowering to 50% pod formation, resulting in excessive levels of lead (Pb) and cadmium (Cd) in mature seeds.^22^

Further analysis of plant physiology indicates that photoperiod significantly influences primary metabolic processes, including photosynthesis and respiration. For example, extended photoperiods can enhance photosynthetic activity by increasing light availability, thereby promoting carbon fixation, while shorter photoperiods may reduce efficiency.^23^ Circadian clock genes synchronize these metabolic activities with daily environmental fluctuations.^24^ Research indicates that photoperiod can regulate the synthesis and distribution of carbohydrates in plants. For example, Potamogeton pectinatus plants grown under long-day conditions allocated less biomass to new tissues than those grown under short-day conditions, resulting in differences in growth and development.^25^

Additionally, changes in photoperiod can influence the fatty acid composition and content in plants’ lipid metabolism. Experiments have shown that the composition of polyphenolic compounds in sweet cherries varies with changes in day length under different photoperiod treatments.^26^ Furthermore, photoperiod can either promote or inhibit the production of specific secondary metabolites. For example, a study on *Saposhnikovia divaricata* showed that photoperiod could impact the accumulation of saikosaponins in the leaves.^27^

Photoperiod, a key environmental signal, universally influences plant stress adaptation. By sensing day length, plants synchronize critical life cycle events, such as flowering, with seasonal changes, enhancing survival and reproductive success under diverse environmental conditions.^28,29^ Photoperiod response genes, such as *Ppd-H1* in barley, interact with drought stress signals to regulate spike development.^30^ Research has shown that changes in photoperiod affect the expression and activity of antioxidant systems, thereby modulating plant antioxidant capacity.^31^ For example, studies conducted in Arabidopsis indicate that in photoperiod stress-sensitive cytokinin receptor mutants (ahk2 ahk3) and clock mutants (cca1 lhy), the redox state of ascorbic acid (AsA) and overall antioxidant capacity are impacted.^32^ Under short-day conditions, losing *AtFtsH4* in Arabidopsis results in altered leaf morphology, increased ROS accumulation, and protein carbonylation,^33^ demonstrating that photoperiod influences a plant’s ability to cope with oxidative stress.

Photoperiod significantly influences mineral absorption and distribution.^34^ Variations in day length alter a plant’s demand for and capacity to absorb minerals. For instance, one study demonstrated that low-intensity, long photoperiod treatment (LILP) enhanced the uptake of most major ions in tomato plants.^35^ In contrast, high-intensity, short photoperiod treatment (HISP) had a relatively weaker effect. Moreover, photoperiod modulates internal mineral transport; research indicates it affects calcium distribution during soybean seed filling.^36^ In tomato seedlings, different photoperiod treatments influence the levels of both macro- and micronutrients.^37^ Additionally, studies in maize have shown a strong interaction between photoperiod and root zone temperature, impacting the concentrations of various mineral elements in both aboveground tissues and roots.^34^

Phytohormones are critical signaling molecules that mediate plant responses to changes in photoperiod.^38^ Research has shown that gibberellin (GA) levels may increase under long days, promoting stem elongation and flowering.^39^ Conversely, elevated abscisic acid (ABA) levels under short-day conditions can induce dormancy or promote tuber formation.^40^ Phytohormones also interact in a complex network to regulate responses to photoperiod.^41^ Auxins, gibberellins, and cytokinins can synergistically promote growth and flowering,^42^ whereas abscisic acid and ethylene typically inhibit growth and induce dormancy or senescence.^43^ Photoperiod modulates the balance among these hormones, thereby directing plant growth and development.^44^

Phytohormones are essential secondary messengers that regulate plants’ heavy metal uptake and translocation. Specifically, hormones such as indole-3-acetic acid (IAA), gibberellins (GA), and salicylic acid (SA) significantly enhance the uptake, translocation, and accumulation of heavy metals.^45^ For instance, combining IAA and EDTA has been shown to improve copper hyperaccumulation in sunflowers,^46^ likely due to hormone-mediated regulation of root morphology, cell membrane permeability, and the expression of related transporter proteins.^47^ Moreover, plant hormones can promote heavy-metal uptake but mitigate toxicity by activating defense mechanisms. Under heavy metal stress, abscisic acid (ABA) levels often increase, triggering signaling pathways that upregulate antioxidant enzymes.^48^ Additionally, brassinosteroids (BRs) – recognized as the sixth class of plant hormones – play crucial roles in growth and development and enhance resistance to abiotic stresses.^49^ Plant hormones also influence physiological processes such as photosynthesis and carbohydrate accumulation, indirectly affecting the plant’s response to heavy metals; for example, exogenous application of GA3 can alleviate the inhibitory effects of heavy metal stress on rice growth and regulate carbohydrate accumulation.^50^

The variation in endogenous hormone levels in *S. alfredii* under different photoperiods remains unclear, limiting its application across diverse regions and hindering the effective use of exogenous hormones across seasons. In this paper, we investigate endogenous hormone levels across a range of photoperiods (0 to 24 h) and examine the corresponding physiological responses, focusing on how photoperiod influences heavy metal uptake and translocation mediated by phytohormones.

## Materials and methods

*S*. *alfredii* seeds, collected from an ancient lead-zinc mining area in Quzhou, Zhejiang Province, were cultivated in a nursery. Then, seedlings of appropriate length were selected and transplanted into pots containing nursery substrate. After 2 months of cultivation, once the seedlings had established stable root systems, the materials with the pot were transferred to illumination incubators for photoperiodic induction treatments. The photoperiod treatments were conducted by exposing *Sedum alfredii* plants to alternating cycles of illumination and darkness in a controlled growth chamber. Specifically, treatments included illumination durations of 2, 4, 6, 8, 10, 12, 14, 16, 18, 20, 22, and 24 h within each 24-h cycle, with corresponding dark periods complementing each illumination period (e.g., 2 h of light followed by 22 h of darkness, 4 h of light followed by 20 h of darkness, and so forth). The light source was provided by white LED lamps (wavelength range: 400–700 nm), with an intensity set at approximately 170.00 µmol m^−2^ s^−1^ photosynthetic photon flux density (PPFD) (Table S1). The PPFD was regularly calibrated using a quantum light meter (OHSP-350SF, Hangzhou, China). Temperature and humidity conditions were maintained consistently throughout all treatments (25°C, ~60% relative humidity). The detailed spectral distribution of the LED lamps used in this study is presented in the supplementary materials (Figure S1). Continuous darkness severely inhibits growth in *Sedum alfredii*, which makes meaningful comparisons challenging; thus, this treatment was not included in our experimental design. For each photoperiod treatment, three spatially independent light blocks were randomly assigned to ensure adequate statistical replication and to minimize spatial variations within the growth chamber. Three spatially independent light blocks were established within the same plant growth room for each photoperiod treatment, each maintained under identical temperature and humidity conditions. One plant was randomly sampled from each block as an independent biological replicate (*n* = 3). Statistical analyses were performed using these replicates, with each light block considered an experimental unit. Photoperiodic induction was carried out for 2 weeks under 25°C to stabilize endogenous hormone levels. Afterward, samples were collected in the dark, rapidly frozen, and stored at −80°C for future analysis.

### Determination of endogenous hormones and flavonoids

Endogenous hormones and flavonoids were quantified according to Park et al.^51^ and Mustafa et al.^52^ with slight modifications. Analytical standards for hormones and flavonoids were obtained from Sigma-Aldrich and Merck (USA). Reagents, including formic acid, methanol, and acetonitrile, were purchased from Fisher Scientific (USA). Approximately 100 mg of each frozen sample was ground and extracted using 80% aqueous methanol, centrifuged, and the resulting supernatant was filtered through 0.22 µm filters before LC-MS/MS analysis. Analyses were performed using a Vanquish UHPLC coupled to a Quantis TSQ mass spectrometer (Thermo Fisher Scientific, USA), operated in electrospray ionization (ESI) and multiple reaction monitoring (MRM) modes. Separation utilized a C18 column at 30°C with gradient elution (10% to 90% acetonitrile with 0.1% formic acid over 5 min at 0.3 mL/min), and mass spectrometric detection was conducted in SRM mode with parameters optimized based on provided Table S1 conditions.

### Determination of key photosynthetic pigments

Chlorophyll a, chlorophyll b, and lutein contents were analyzed according to the method described by Zeb,^53^ with slight modifications. Analytical standards were purchased from Sigma–Aldrich (USA), and all solvents, including formic acid, methanol, and acetonitrile, were obtained from Fisher Scientific (USA). Approximately 100 mg of frozen plant material was ground and extracted with 5 mL of ice-cold acetone by orbital shaking at 130 rpm for 60 min, then 10 mL of absolute ethanol and vortexing for 30 min. The extraction was repeated until residues became colorless, and solvents were evaporated under vacuum at 35°C. The residues were re-dissolved in 2 mL of methanol and filtered through 0.45 µm PTFE filters before analysis. HPLC analyses were performed using a 2695 HPLC system coupled with a Photo-Diode Array Detector (PDA, Waters, USA). Chromatographic separation was achieved on a C18 column maintained at 25°C using a ternary gradient mobile phase of methanol, water, and methyl tertiary butyl ether (MTBE). Detection wavelengths were set at 450 nm for carotenoids and 650 nm for chlorophylls, with identification confirmed by comparing retention times and spectral characteristics against standards.

### Determination of gene expression

Hormone metabolism and gene expression related to Cd response were measured by qPCR.^54^ Total RNA was extracted using a Plant Total RNA Isolation Kit (Sangon, China), and its quality and quantity were assessed with a NanoDrop microspectrophotometer (Thermo Fisher Scientific, USA). First-strand cDNA was synthesized from 1 μg of total RNA using the MightyScript First Strand cDNA Synthesis Master Mix (Sangon). Quantitative real-time PCR (qPCR) was then performed on a CFX Opus Real-Time PCR System (Bio-Rad, USA) with 2X SYBR Green Abstart PCR Mix (Sangon). Each of these kits was used according to the manufacturer’s protocols. The amplification program included an initial denaturation at 95°C for 10 min, followed by 60 cycles of 95°C for 10 s, annealing at the primer-specific Tm for 10 s, and extension at 72°C for 15 s. A melting curve analysis was conducted from 65°C to 95°C to confirm primer specificity. Each experiment was performed with three biological replicates and three technical replicates. Primer sequences used for gene expression analysis are listed in Supplementary Table S2.

### Statistics and analysis

One-way analysis of variance (ANOVA) was performed using SPSS (IBM, USA), and plots were drawn using Origin (OriginLab, USA). Correlation analysis and heat maps were generated using R packages.

## Result

### Endogenous hormone content under different photoperiods

Under various photoperiod treatments, endogenous hormone levels in *S. alfredii* exhibited distinct fluctuations (Table 1). In this context, the reported hours refer to the duration of light exposure. Abscisic acid (ABA) levels increased significantly, peaking at 6 h (6.43 µg/g) before decreasing, suggesting a transient accumulation response to prolonged light exposure. ACC levels continuously declined from a high initial concentration at 2 h (13,566.4 µg/g) to a lower level at 6 h (6,685.1 µg/g) before slightly rebounding under extended light exposure. Brassinosteroid (Br) concentrations peaked sharply at 4 hs (11.79 µg/g) and then stabilized at moderate levels, reflecting a possible early-stage growth regulation response. Gibberellin (GA3) levels steadily increased across the photoperiod treatments, rising from 2.5 µg/g at 2 h to 4.5 µg/g at 10 h, indicating a gradual accumulation over time.

**Table 1.** The changes in endogenous hormones of *Sedum alfredii* Hance under different photoperiods.

	ABA	ACC	Br	GA3	IAA	JA	MeJA	SA	SL	tZ
2 h	0.17 ± 0.00k	13656.84 ± 45.22a	4.89 ± 0.02 g	2.53 ± 0.05i	0.86 ± 0.00f	0	10.95 ± 0.14a	0	1129.58 ± 18.22d	1022.03 ± 5.84a
4 h	1.87 ± 0.04	7830.98 ± 91.97e	12.02 ± 0.18a	3.26 ± 0.05gh	1.20 ± 0.02b	0	0	21.43 ± 0.25a	1537.60 ± 8.97a	22.39 ± 0.34de
6 h	6.44 ± 0.02a	6707.40 ± 58.96f	7.62 ± 0.07d	3.74 ± 0.06f	1.37 ± 0.01a	0	0	9.18 ± 0.08d	0	50.58 ± 0.29b
8 h	4.52 ± 0.04c	8580.86 ± 150.35c	9.23 ± 0.16c	4.13 ± 0.05d	1.16 ± 0.02b	0	0	5.30 ± 0.06e	0	19.08 ± 0.31ef
10 h	1.22 ± 0.01f	9631.85 ± 139.48b	9.78 ± 0.085b	4.46 ± 0.07c	0.96 ± 0.01de	6.66 ± 0.04a	0	10.55 ± 0.16b	0	51.66 ± 0.67b
12 h	0.83 ± 0.01 h	8079.56 ± 233.24de	7.41 ± 0.11d	4.86 ± 0.03a	0.8 ± 0.00 g	0	0	9.48 ± 0.05c	0	37.60 ± 0.22c
14 h	1.25 ± 0.02f	8574.42 ± 247.52c	5.68 ± 0.05e	4.65 ± 0.10b	0.86 ± 0.02f	0	0	0.72 ± 0.01i	1265.35 ± 8.38c	23.07 ± 0.20de
16 h	0.50 ± 0.01j	8377.42 ± 125.46 cd	5.37 ± 0.15f	4.26 ± 0.02d	0.89 ± 0.01f	0	0	1.58 ± 0.01 g	0	27.98 ± 0.25d
18 h	1.04 ± 0.02 g	8563.65 ± 176.50c	3.95 ± 0.06 h	3.92 ± 0.06e	0.88 ± 0.02f	0	0	1.02 ± 0.02 h	0	26.92 ± 0.44d
20 h	3.73 ± 0.04d	8091.85 ± 120.00de	3.87 ± 0.06 h	3.72 ± 0.09f	0.94 ± 0.01e	0	10.34 ± 0.12b	3.88 ± 0.05f	1351.57 ± 4.52b	16.34 ± 0.25f
22 h	5.22 ± 0.02b	6929.98 ± 69.99f	2.86 ± 0.05i	3.35 ± 0.03 g	1.06 ± 0.02c	0	0	1.75 ± 0.02 g	0	25.84 ± 0.53d
24 h	0.61 ± 0.01i	7940.63 ± 93.87de	2.01 ± 0.02j	3.1 ± 0.05 h	1.00 ± 0.01d	0	0	0.90 ± 0.01hi	0	24.19 ± 0.56de

Values represent three biological replicates’ mean ± standard error (SE). Different lowercase letters within a column indicate significant differences among varieties (ANOVA, *p* < 0.05). The same lettering convention is used in subsequent tables.

Indoleacetic acid (IAA), which was included in the current dataset, exhibited a clear peak at 4 h (2.1 µg/g) before sharply declining and stabilizing at lower levels in subsequent treatments. This pattern suggests an early, light-induced activation of auxin biosynthesis, potentially linked to initial growth responses.

Salicylic acid (SA) exhibited a pronounced peak at 4 h (20.94 µg/g) before declining, indicating a potential role in early stress signaling. Strigolactone (SL) was initially high (1,093.14 µg/g) at 2 h but sharply declined after 4 h, becoming undetectable in subsequent treatments. Finally, trans-zeatin (tZ) showed a sharp peak at 2 h (1,011.91 µg/g) before rapidly decreasing, followed by moderate fluctuation in later stages.

These results indicate that hormone responses in *S. alfredii* are highly time-dependent and exhibit unique regulatory patterns under varying photoperiod durations, reflecting diverse physiological roles in growth and stress adaptation.

### Flavonoid content under different photoperiods

Under different photoperiod treatments, the concentrations of flavonoid compounds in *S. alfredii* exhibited apparent temporal variations (Table 2). Quercetin peaked at 6 h of light exposure, indicating a transient upregulation at this time point, followed by a rapid decline. Astragalin levels gradually increased, peaking at 10 h, which suggests delayed biosynthesis. Chlorogenic acid was mainly undetectable throughout the photoperiods, with only trace amounts occasionally observed, reflecting limited accumulation under the tested conditions. Phenolic acid increased initially, reaching a peak at 6 h before declining to lower levels. Rutin maintained relatively high and stable concentrations between 4 and 10 h, signifying sustained accumulation during the middle stages of light exposure. Rosmarinic acid accumulated significantly from 8 h and remained high until 10 h. In contrast, salvianolic acids A and B and sinapic acid were either undetectable or present only at negligible levels across all time points, implying minimal biosynthesis or accumulation under these conditions. Overall, these findings demonstrate that flavonoid compounds exhibit distinct accumulation patterns in response to photoperiod variations, suggesting temporal specificity in the metabolic regulation of *S. alfredii*.

**Table 2.** The changes in flavonoid of *Sedum alfredii* Hance under different photoperiods.

	Astragalin	Caffeic Acid	Cinnamic acid	Coumalic acid	Phenolic Acid	Quercetin	Quinic acid	Rosmarinic Acid	Rutin	Salvianolic Acid A
2 h	0	1.45 ± 0.02e	1379.28 ± 39.82i	0	0	0	312.79 ± 1.04e	0	0	0
4 h	0	0	139772.28 ± 815.13 h	835.48 ± 21.82a	6.07 ± 0.10ef	0	223.92 ± 4.56f	0	42.58 ± 0.50e	0
6 h	0.16 ± 0.00 h	1.25 ± 0.00f	392828.46 ± 3851.26d	787.92 ± 10.79bcd	13.06 ± 0.00d	204.22 ± 4.29a	98.93 ± 1.30 h	0	48.36 ± 0.75 cd	0
8 h	0	5.35 ± 0.08a	560898.40 ± 13000.96a	749.30 ± 8.83d	6.49 ± 0.11e	0	443.41 ± 6.28b	102.54 ± 1.60b	46.10 ± 0.27 cd	0
10 h	0.70 ± 0.01d	0	452146.58 ± 10620.89c	747.08 ± 2.48d	2.90 ± 0.045 g	0	74.73 ± 0.67i	106.37 ± 1.74a	55.1 ± 1.27a	0
12 h	1.02 ± 0.01b	0.74 ± 0.02 h	312097.14 ± 2761.64 g	772.22 ± 11.45 cd	7.70 ± 0.03 cd	206.64 ± 5.97a	111.08 ± 1.68 h	0	47.25 ± 0.71 cd	0
14 h	0.99 ± 0.01c	2.01 ± 0.02d	404941.37 ± 2717.73d	760.39 ± 8.93 cd	15.26 ± 0.18b	0	150.40 ± 1.84 g	0	48.42 ± 0.72 cd	0
16 h	0.51 ± 0.00f	4.42 ± 0.05b	486789.65 ± 5811.74b	763.10 ± 8.81 cd	22.53 ± 0.34a	0	336.65 ± 5.52d	0	48.16 ± 0.42 cd	160.96 ± 1.0a
18 h	0.53 ± 0.01e	3.13 ± 0.01c	455525.46 ± 7028.55c	769.93 ± 6.88 cd	9.64 ± 0.14 cd	210.98 ± 2.48a	867.54 ± 13.52a	0	46.01 ± 0.74d	0
20 h	1.40 ± 0.02a	0	358029.57 ± 6734.54e	790.91 ± 20.66bc	6.40 ± 0.07e	0	370.93 ± 7.50c	0	48.51 ± 1.12c	0
22 h	0.41 ± 0.01 g	1.06 ± 0.01 g	337555.88 ± 5021.75f	814.89 ± 16.58ab	13.73 ± 0.23c	208.51 ± 2.06a	57.08 ± 0.86j	0	52.49 ± 0.93b	0
24 h	0.69 ± 0.01d	0.69 ± 0.01 h	397980.39 ± 3498.20d	838.62 ± 12.56a	5.89 ± 0.04f	0	223.46 ± 1.28f	0	53.51 ± 0.53ab	0

Chlorogenic Acid, protamine sulfate, salvianolic acid A, and sinapic Acid were not detected.

### The expression of the primary hormone metabolism gene under different photoperiods

**Figure 1.**
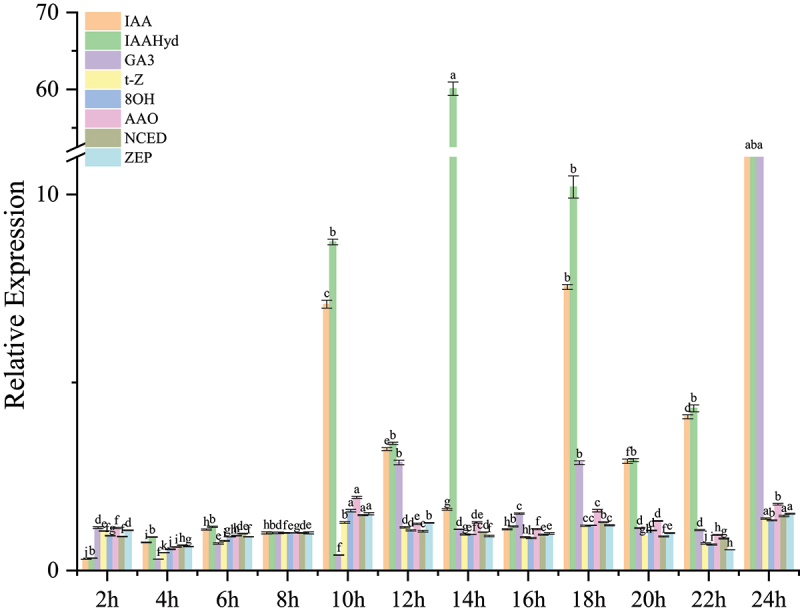
The changes in expression levels of endogenous hormone metabolic genes in *Sedum alfredii* under different photoperiods.

The analysis of the effects of varying photoperiod durations on endogenous hormone metabolism enzymes in *S. alfredii* revealed distinct regulatory patterns (Figure 1). For auxin metabolism, the synthetic enzyme IAA exhibited low initial expression levels under short light exposure (2–6 hs), but its expression dramatically increased after 8 h, peaking sharply at 10 h. Similarly, the auxin degradation enzyme (IAAHyd) followed this pattern, suggesting enhanced auxin turnover with more prolonged light exposure. Gibberellin biosynthesis (GA3) showed relatively stable expression initially but decreased notably at 4 and 10 h, indicating potential suppression under prolonged light conditions. The cytokinin biosynthesis enzyme (t-Z) remained relatively constant, with only a moderate increase at 10 h. ABA biosynthetic enzymes (AAO, NCED, ZEP) exhibited a similar trend: their expression decreased at a short photoperiod (4 h) and then progressively increased, peaking at 10 h. In contrast, the ABA degradation enzyme (8OH) mirrored the ABA synthetic enzymes’ patterns, with higher expression observed under extended light exposure (8–10 h), indicative of active ABA turnover. These results suggest that longer photoperiods significantly enhance auxin and ABA metabolism, synthesis, and degradation while exerting suppressive effects on gibberellin biosynthesis in *S. alfredii*.

Bar heights indicate the mean values of three biological repetitions. Error bars represent the standard error of the mean (SE). Different lowercase letters above the bars indicate significant differences among varieties (ANOVA, *p* < 0.05); the same lettering convention is used in subsequent figures.

### The expression of the resistant enzyme gene under different photoperiods

**Figure 2.**
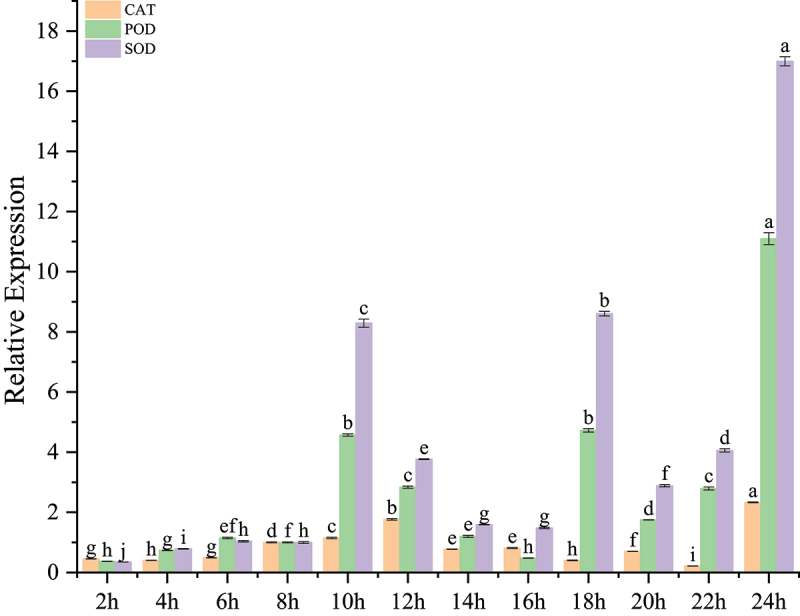
The changes in expression levels of resistant enzyme gene in *Sedum alfredii* under different photoperiods.

The influence of varying photoperiod durations on endogenous resistance enzyme activities in *S. alfredii* showed distinct, time-dependent patterns (Figure 2). Under photoperiod treatments, catalase (CAT) activity remained stable from 2 to 6 h of light exposure, then increased significantly at 8 h, peaking around 10 h. Similarly, peroxidase (POD) activity gradually increased from 2 to 6 h, sharply rising after 8 h and reaching a maximum at 10 h, indicating a robust oxidative stress response. Likewise, superoxide dismutase (SOD) exhibited a moderate increase up to 6 h, followed by a sharp rise between 8 and 10 h, with the highest activity observed at 10 h. These findings suggest that extended light exposure enhances the activities of these antioxidant enzymes, highlighting their potential roles in photoprotection mechanisms in *S. alfredii*.

### The expression of the heavy-metal accumulation gene under different photoperiods

Under varying photoperiod treatments, significant fluctuations in the expression levels of heavy metal transporter genes were observed in *S. alfredii* (Table 3). Notably, *ZIP* family members demonstrated distinct patterns; *ZIP1* exhibited peak expression at 10 h photoperiod, reaching up to approximately 17-fold and 7.7-fold, respectively, compared to the control. *ZIP2* peaked later, at 24 h photoperiod, suggesting delayed induction under extended photoperiod conditions. Among *HMA* transporters, *HMA4* expression drastically increased at 10 h, showing nearly a 60-fold enhancement, whereas *HMA2* and *HMA3* showed modest increases, peaking at 6 h and 24 h photoperiod, respectively. *MT2* expression peaked at 20 h photoperiod, indicating its involvement in late-stage photoperiod responses. *Nramp* family transporters displayed lower but consistent variation, with *Nramp6* reaching its highest expression at 14 h photoperiod. These results indicate transporter-specific responses to photoperiod duration, highlighting distinct temporal regulation and suggesting potential adaptive mechanisms for heavy metal uptake under varying light conditions.

**Table 3.** The changes in expression levels of heavy-metal transporter gene in *Sedum alfredii* under different photoperiods.

	ZIP1	ZIP2	ZIP3	HMA2	HMA3	HMA5	MT2	Nramp4	Nramp5	Nramp1	Nramp6
2 h	0.77 ± 0.01 g	0.16 ± 0.00i	0.97 ± 0.01fg	0.48 ± 0.01j	0.68 ± 0.00f	1.33 ± 0.02k	0.50 ± 0.01 h	0.47 ± 0.01 g	1.42 ± 0.03c	0.86 ± 0.00d	0.97 ± 0.01d
4 h	0.99 ± 0.01f	0.81 ± 0.01 g	1.12 ± 0.01b	0.015 ± 0.00k	0.25 ± 0.00j	3.66 ± 0.05i	1.63 ± 0.02e	0.72 ± 0.00e	0.95 ± 0.01 g	0.47 ± 0.01 h	0.22 ± 0.00 h
6 h	1.08 ± 0.02f	0.89 ± 0.00 g	1.12 ± 0.02bc	1.97 ± 0.06a	0.74 ± 0.01e	0.34 ± 0.00 l	1.07 ± 0.02 g	0.58 ± 0.00f	1.08 ± 0.01e	0.66 ± 0.00f	0.27 ± 0.01 g
8 h	1 ± 0.01f	1 ± 0.01f	1 ± 0.02ef	1 ± 0.01e	1 ± 0.02c	1 ± 0.03kl	1 ± 0.02 g	1 ± 0.03c	1 ± 0.01f	1 ± 0.03b	1 ± 0.01d
10 h	7.57 ± 0.09a	0.47 ± 0.00 h	1.31 ± 0.03a	0.76 ± 0.02 g	0.40 ± 0.00 h	58.10 ± 0.87a	1.60 ± 0.02e	1.02 ± 0.01c	0.36 ± 0.01 h	1.08 ± 0.02a	0.14 ± 0.00i
12 h	2.06 ± 0.02e	2.05 ± 0.02d	1.00 ± 0.01ef	1.48 ± 0.02b	0.33 ± 0.01i	24.75 ± 0.30b	1.30 ± 0.01f	1.28 ± 0.03b	1.49 ± 0.01b	0.98 ± 0.01b	0.31 ± 0 g
14 h	2.22 ± 0.03d	3.86 ± 0.06b	0.93 ± 0.01 g	1.30 ± 0.02c	0.81 ± 0.00d	11.16 ± 0.07e	1.45 ± 0.03ef	0.57 ± 0.01f	1.18 ± 0.02d	0.92 ± 0.01c	1.91 ± 0.03a
16 h	2.07 ± 0.03e	2.63 ± 0.05c	1.05 ± 0.01de	0.90 ± 0.01f	0.80 ± 0.02d	14.30 ± 0.41c	2.89 ± 0.02d	0.1 ± 0.00i	1.14 ± 0.02d	0.75 ± 0.01e	1.11 ± 0.02b
18 h	2.55 ± 0.03c	1.17 ± 0.02e	1.09 ± 0.01bcd	0.55 ± 0.014i	0.65 ± 0.01fg	6.67 ± 0.10 g	4.57 ± 0.055c	1.66 ± 0.02a	1.14 ± 0.01d	0.88 ± 0.02 cd	1.05 ± 0.02c
20 h	2.28 ± 0.03d	1.17 ± 0.02e	0.99 ± 0.02f	0.79 ± 0.001 g	1.31 ± 0.02b	4.68 ± 0.07 h	13.19 ± 0.20a	1.26 ± 0.01b	1.66 ± 0.03a	0.61 ± 0.01 g	0.15 ± 0.00i
22 h	2.10 ± 0.02e	1.12 ± 0.01e	0.85 ± 0.02 h	0.63 ± 0.01 h	0.64 ± 0.01 g	7.60 ± 0.11f	0.20 ± 0.00i	0.93 ± 0.02d	1.15 ± 0.01d	0.59 ± 0.01 g	0.91 ± 0.02e
24 h	5.76 ± 0.03b	5.42 ± 0.10a	1.06 ± 0.02 cd	1.0758 ± 0d	1.37 ± 0.02a	13.11 ± 0.16d	5.3 ± 0.09b	0.28 ± 0.01 h	0.03 ± 0.00i	1.06 ± 0.02a	0.67 ± 0.01f

### The pigment content under different photoperiods

**Figure 3.**
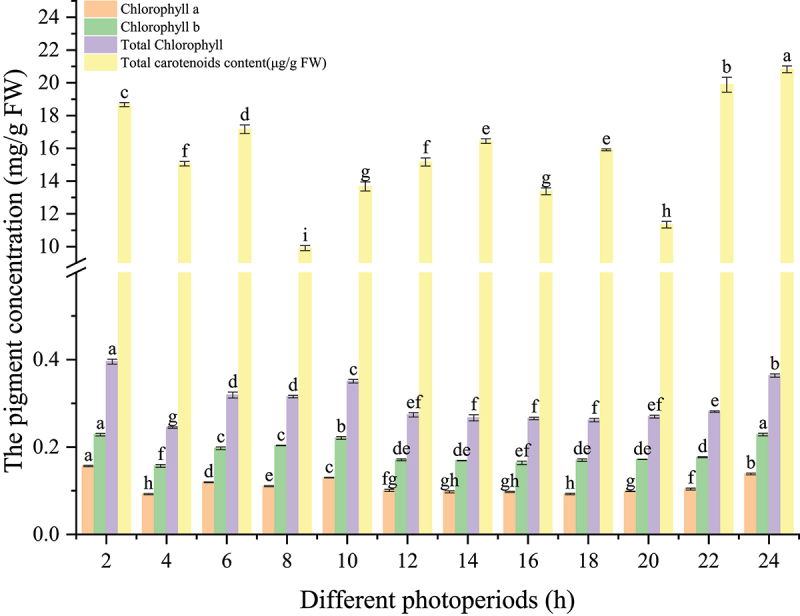
Variations in photosynthetic pigments of *Sedum alfredii* exposed to differential photoperiods.

The pigment content in Sedum varied significantly in response to the photoperiod, as indicated by the changes in chlorophyll and carotenoid levels (Figure 3). Chlorophyll a content ranged from 0.0923 mg/g FW to 0.1567 mg/g FW, with the highest concentration observed at 2 h of light exposure (0.1567 mg/g FW), significantly higher than all other time points (*p* < 0.05). Conversely, the Chlorophyll a content was lowest at 4 h of light exposure (0.0923 mg/g FW), showing a significant reduction compared to other durations (*p* < 0.05).

Similarly, chlorophyll b content exhibited a downward trend as exposure time increased, peaking at 2 h (0.2283 mg/g FW) and steadily decreasing to 0.1567 mg/g FW by 4 h (Sa_4h). The total chlorophyll content followed the same pattern, with the highest levels recorded at 2 h (0.3955 mg/g FW), decreasing to 0.2446 mg/g FW at 4 h. This reduction was statistically significant compared to the early time points (*p* < 0.05).

Carotenoid content showed a distinct trend, with the highest values recorded at 2 h of light exposure (18.6684 μg/g FW) and a steady decline at subsequent time points. Notably, carotenoid content was lowest at 8 h (Sa_8h, 9.9133 μg/g FW), demonstrating a statistically significant reduction from the earlier time points (*p* < 0.05).

### CCA analysis of distinct hormone – pigment – gene modules

**Figure 4.**
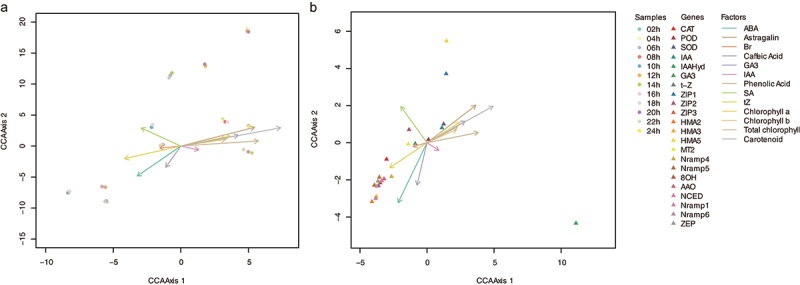
Canonical correspondence analysis of photoperiod-induced shifts in hormone signaling, pigment dynamics, and Cd transporter expression.
Biplot of individual sample replicates and photo-responsive substance profiles.Biplot of photo-responsive substance profiles and Cd accumulation gene variables. Sample points represent biological replicates, while each gene point indicates the overall loading of that gene in the CCA, not replicate values. Vectors show variable associations with the canonical axes.

The CCA analysis integrating endogenous hormone levels, pigment contents (environmental variables), and Cd transporter gene expression (response variables) revealed distinct spatial distribution patterns for different photoperiod treatments, highlighting clear gradients along the primary CCA axes (Figure 4). The first two canonical axes accounted for 71.3% (CCA1) and 18.7% (CCA2) of the total variance, respectively, capturing the principal directions of variation in the combined dataset.

Samples exposed to shorter photoperiods (e.g., 02 h to 08 h) were primarily positioned along the negative regions of both CCA1 and CCA2, closely aligning with the vectors for ABA (abscisic Acid), tZ (trans-zeatin), and SA (salicylic acid). This indicates a strong association with stress-related hormonal responses under shorter light exposure, consistent with early-stage defense activation and growth suppression. In contrast, samples from longer photoperiods (e.g., 20 h to 24 h) clustered in the positive quadrants of CCA1 and CCA2, corresponding to vectors for Chlorophyll, carotenoids, Astragalin, and GA₃ (gibberellic acid), reflecting enhanced photosynthetic activity and growth-promoting signals typical of extended light exposure.

Intermediate photoperiods (10 h to 16 h) occupied transitional positions, reflecting a blend of stress adaptation and growth stimulation. Notably, samples at 10 h, which previously demonstrated peak *HMA4* and *ZIP1* expression, were located near the intersection of IAA (indole-3-acetic acid) and SA vectors, suggesting a complex hormonal signaling state balancing growth and defense responses. This is consistent with the observed upregulation of antioxidant enzyme activities and heavy metal transporter expression at this photoperiod.

The distribution of samples along the CCA axes underscores the critical role of photoperiod in modulating hormonal and pigment landscapes in *S. alfredii*, directly impacting Cd uptake and detoxification processes. Short photoperiod samples aligning with ABA and SA vectors likely reflect early stress adaptation mechanisms, including oxidative stress mitigation and growth inhibition. In contrast, long photoperiod samples associated with chlorophyll and GA₃ vectors indicate a strategic shift toward photosynthetic enhancement and biomass accumulation, optimizing growth under extended light conditions.

These spatial patterns highlight the potential for photoperiod manipulation to fine-tune hormone-pigment dynamics in *S. alfredii*, potentially enhancing Cd uptake efficiency under controlled cultivation. Such insights provide a foundation for optimizing phytoremediation strategies by synchronizing transporter activation with peak photosynthetic activity, maximizing metal uptake while minimizing oxidative damage.

These findings emphasize photoperiod-induced responses’ complex, multivariate nature in hyperaccumulator plants, reflecting a dynamic interplay between light exposure, hormone signaling, and heavy metal tolerance. Future studies should explore the molecular mechanisms underlying these interactions to refine photoperiod-based cultivation protocols for enhanced phytoremediation performance.

## Discussion

### Photoperiod effects on endogenous hormone levels

Photoperiod manipulation significantly influenced the endogenous hormone profiles of *S. alfredii*, highlighting day length as a critical regulator of growth and stress signaling pathways. Under short-day conditions, abscisic acid (ABA) levels exhibited a pronounced, transient peak at 6 h before declining, mirroring responses observed in *Medicago sativa*, where short photoperiods promote dormancy through ABA accumulation mediated by phytochrome B (PhyB).^55^ Similar ABA dynamics are reported in woody perennials, where short-day conditions trigger growth cessation, bud formation, and dormancy.^56^ This rapid, transient ABA response likely represents an early stress adaptation mechanism that primes the plant for reduced photosynthetic capacity and environmental stress.

In contrast, Gibberellin (GA) biosynthesis was suppressed under prolonged light exposure, reflecting a dual photoreceptor-mediated regulatory mechanism. In rice, PhyB represses the expression of GA biosynthesis genes (GA20ox, GA3ox), while cryptochrome 1 (cry1) simultaneously upregulates inactivation genes (GA2ox), together ensuring robust GA suppression under white light to optimize seedling architecture for dense cultivation.^57,58^ This coordinated repression likely prevents excessive stem elongation under extended photoperiods, aligning plant morphology with competitive light environments.

In addition to ABA and GA, salicylic acid (SA) and jasmonic acid (JA) showed distinct temporal patterns. SA levels spiked rapidly under short (4-h) photoperiods, likely initiating early stress responses, while JA accumulated under longer (10-h) exposures, consistent with its role in sustained defense signaling in Arabidopsis.^59^ This temporal partitioning suggests that SA acts as an immediate stress signal,^60^ while JA contributes to longer-term adaptive responses to prolonged light exposure.^61^ Brassinosteroids (BRs) also peaked early (4 h), aligning with their established roles in rapid growth signaling and early photomorphogenic responses.^62^

Indole-3-acetic acid (IAA) levels increased progressively with photoperiod length, suggesting that extended light exposure stimulates IAA biosynthesis or reduces its degradation.^63^ This trend is consistent with IAA’s central role in cell elongation, division, and organogenesis, key processes for optimizing growth under favorable light conditions. The gradual accumulation of IAA under extended photoperiods likely reflects a strategic shift toward enhanced growth, supporting increased photosynthetic capacity and resource capture as light availability improves.

Together, these results demonstrate that *S. alfredii* dynamically adjusts its hormone balance in response to changing photoperiods, transitioning from early stress signaling to sustained growth promotion. This photoperiod-induced hormonal plasticity offers a precise, non-chemical approach to modulate plant resilience, presenting a promising strategy for enhancing stress tolerance and optimizing biomass production in controlled cultivation systems.

### Photoperiodic regulation of cadmium transporter-related genes

Photoperiod duration significantly influenced the transcriptional landscape of cadmium (Cd) transport and detoxification in *Sedum alfredii*. Moderate photoperiods (8 to 10 h) strongly induced *ZIP1* and *HMA4*, critical genes for Cd uptake and xylem loading.^64^ Under extended light conditions, sustained expression of *ZIP2*, *HMA3*, and *MT2* further suggests activation of additional pathways for Cd sequestration and detoxification.^65^ *MT2* encodes a cysteine-rich metallothionein that binds Cd, facilitating long-term detoxification.^66^ This photoperiod-dependent transcriptional activation indicates a tiered regulatory response, where downstream detoxification mechanisms complement early-stage transport under prolonged light exposure.

Antioxidant enzyme activities, including catalase (CAT), peroxidase (POD), and superoxide dismutase (SOD), also peaked under 10-h photoperiods, aligning with findings in Arabidopsis thaliana where extended light triggers reactive oxygen species (ROS) production and corresponding antioxidant defenses.^67^ These enzymes play crucial roles in mitigating oxidative damage by scavenging ROS generated during photosynthesis and heavy metal stress. The synchronous upregulation of antioxidant enzymes and Cd transporter genes suggests a coordinated response, allowing plants to efficiently manage metal uptake and oxidative stress.

Moreover, the overlapping peaks of jasmonic acid (JA) and antioxidant enzyme activity under similar photoperiods indicate tight hormonal control over stress responses. This aligns with previous studies where exogenous salicylic acid (SA) and abscisic acid (ABA) treatments enhanced Cd tolerance in *S. alfredii* by boosting antioxidant capacity and stress-related gene expression.^68,69^ Together, these results highlight the complex interplay between photoperiod, metal transport, and oxidative stress management, emphasizing the potential for light duration as a modulator of heavy metal tolerance.

### Photoperiod-driven pigment dynamics and oxidative stress responses

The observed decline in pigment content (Chlorophyll and carotenoids) with increasing photoperiod in *Sedum alfredii* presents an intriguing deviation from the typical trend seen in many higher plants, where prolonged light exposure often promotes pigment accumulation. This inverse relationship likely reflects a species-specific adaptation to balance light capture efficiency and photoprotection, particularly relevant for a hyperaccumulator species like *S. alfredii* that frequently encounters oxidative stress.

Prolonged light exposure can lead to photoinhibition and photodamage, directly impacting pigment stability. Under extended photoperiods, photosystems are continually excited, potentially overwhelming the dark reactions of photosynthesis, which rely on CO_2_ assimilation to utilize the absorbed energy. This imbalance can generate excessive reactive oxygen species (ROS) within the chloroplast, damaging chlorophyll molecules and disrupting membrane integrity.^70^ Additionally, over-excitation of PSII can cause the D1 protein to degrade, further impairing light-harvesting capacity and accelerating pigment loss.^71^ In *S. alfredii*, this phenomenon is evident in the significant pigment decline observed at 4 h and beyond, likely reflecting an early onset of photodamage as light duration increases.

The observed pattern of declining pigment levels aligns well with the increased antioxidant enzyme activities (SOD, POD, CAT) detected under extended photoperiods (8–10 h). These enzymes are critical for mitigating oxidative stress by scavenging ROS, including superoxide radicals and hydrogen peroxide, which can directly degrade Chlorophyll and carotenoids.^72,73^ This suggests that as photoperiod lengthens, *S. alfredii* experiences a significant oxidative burden, forcing the plant to divert resources from pigment synthesis toward ROS detoxification, thereby reducing pigment content. Furthermore, consuming carotenoids as precursors for abscisic acid (ABA) under stress conditions may also reduce their levels.^74^

ABA and JA are critical in regulating pigment turnover under stress. In *S. alfredii*, ABA levels peaked at 6 h, corresponding with the initial decline in Chlorophyll and carotenoids, potentially signaling early stress adaptation. ABA activates chlorophyll degradation pathways and senescence-related genes, promoting pigment breakdown as a protective mechanism.^74^ Similarly, JA, which accumulated at 10 h, is associated with chlorophyll degradation and leaf senescence, further supporting the observed pigment reduction at prolonged light exposures.^75^ These findings suggest that the concurrent increase in ABA and JA under long photoperiods accelerates pigment turnover to mitigate photodamage and oxidative stress.

Finally, the unique pigment response in *S. alfredii* may reflect an adaptive strategy for surviving in high-stress environments. As a hyperaccumulator, *S. alfredii* must manage heavy metal stress and high light exposure, potentially prioritizing antioxidative defenses over pigment retention. This study’s elevated antioxidant enzyme activities indicate a shift from light harvesting to photoprotection under extended photoperiods, allowing the plant to maintain cellular integrity in harsh conditions.^76^ This trade-off likely reflects an evolved resilience mechanism, optimizing survival at the cost of pigment density.

In summary, the photoperiod-dependent pigment dynamics in *S. alfredii* are likely driven by photodamage, ROS accumulation, and stress hormone signaling. These factors collectively reduce pigment synthesis and accelerate degradation, reflecting a species-specific strategy to balance growth and stress tolerance. Understanding these mechanisms is crucial for optimizing *S. alfredi*i’s use in phytoremediation, where maintaining photosynthetic efficiency under variable light conditions is critical.

Despite our comprehensive analysis, there are several limitations in the present study that should be acknowledged. First, while we have extensively investigated hormonal profiles and gene expression patterns under varying photoperiods, the actual Cd accumulation in plant tissues was not measured. This absence limits our ability to directly correlate gene expression changes with Cd uptake efficiency and tolerance mechanisms.

Second, the current experiments were conducted in controlled laboratory conditions, and it remains uncertain how these findings would translate into real field conditions where plants face complex environmental variables such as varying soil types, nutrient availability, microbial interactions, and climatic stresses.

### Modular hormone – pigment signaling governs photoperiod-dependent Cd uptake

Our CCA results indicate that light duration orchestrates a finely tuned hormonal and pigment landscape that, in turn, directs the expression of key Cd transporters in *S. alfredii*. The near-perfect alignment of IAA monooxygenase (*IAAHyd*) with IAA underscores auxin’s pivotal role in preparing root tissues for metal uptake, likely through enhanced lateral root proliferation and cell wall remodeling.^77^ This relationship aligns well with the established function of IAA as a primary regulator of root architecture and nutrient acquisition.

Concurrently, the co-orientation of *HMA4* and *ZIP1* with ABA and caffeic acid vectors suggests that ABA-mediated stress signaling amplifies heavy-metal transporter transcription, consistent with previous findings that ABA enhances HMA expression under Cd challenge.^68^ This co-regulation indicates a broader role for ABA in modulating metal tolerance by integrating oxidative stress signals with heavy metal detoxification pathways.

Interestingly, *HMA2*, *HMA3*, and *NRAMP6* exhibited an inverse relationship to flavonoid (astragalin) and chlorophyll a levels. Flavonoids are known to chelate Cd and mitigate oxidative damage,^78^ suggesting a potential trade-off between pigment-driven metal chelation and transporter activity. The positive alignment of these genes with trans-zeatin (tZ) implies that cytokinin signals may offset flavonoid-mediated suppression, promoting transporter expression during active cell division – a mechanism observed in hyperaccumulators to sustain metal flux under oxidative stress.^79^ This balance between cytokinin stimulation and flavonoid inhibition likely reflects a finely tuned response to fluctuating oxidative stress conditions.

The clustering of these regulatory axes around the 10-h light treatment suggests that midday illumination synchronizes peak hormone and pigment signals, maximizing transporter gene induction. This temporal coordination aligns with the circadian regulation of metal transporter transcripts, which optimize nutrient and metal uptake during periods of highest photosynthetic activity.^80,81^

The spatial distribution of samples along the CCA axes, reflecting their alignment with specific hormone and pigment gradients, highlights the critical influence of photoperiod in shaping the physiological responses of *S. alfredii*. Short photoperiod samples closely associated with ABA and SA vectors likely reflect early stress adaptation mechanisms, aligning with the known roles of these hormones in oxidative stress management and growth suppression.^82,83^ In contrast, the clustering of long photoperiod samples near vectors for Chlorophyll, GA₃^84^, and Astragalin underscores the shift toward photosynthetic enhancement and biomass accumulation under extended light conditions.^84^ This differential alignment suggests that photoperiod impacts transporter gene expression and directly influences broader metabolic networks, including pigment biosynthesis and stress signaling.

Additionally, intermediate photoperiod samples (e.g., 10 h) positioned near the intersection of IAA and SA vectors indicate a complex hormonal environment characterized by simultaneous growth and defense signaling.^85^ This is consistent with the observed upregulation of antioxidant enzymes and Cd transporter expression at this critical photoperiod, reflecting a strategic balance between resource allocation and stress mitigation.^86^

Together, these findings suggest that a comprehensive understanding of sample–environment interactions is crucial for optimizing phytoremediation strategies, as it allows for precise tuning of photoperiod conditions to enhance metal uptake efficiency while minimizing oxidative damage. Future studies should aim to validate these spatial patterns at the molecular and physiological levels, potentially integrating transcriptomic and metabolomic data to reveal the underlying regulatory mechanisms.

By mapping these CCA-derived correlations onto established signaling pathways, our findings propose a model in which optimized light schedules can enhance phytoextraction efficiency by synchronizing transporter activation with peak nutrient uptake periods. Future studies should aim to validate these multivariate associations at the physiological level, including measurements of root architecture, hormone flux, and actual Cd accumulation, as well as promoter-level interactions between light receptors, hormone biosynthetic genes (e.g., *NCED* for ABA), and transporter loci.

## Conclusion

**Figure 5.**
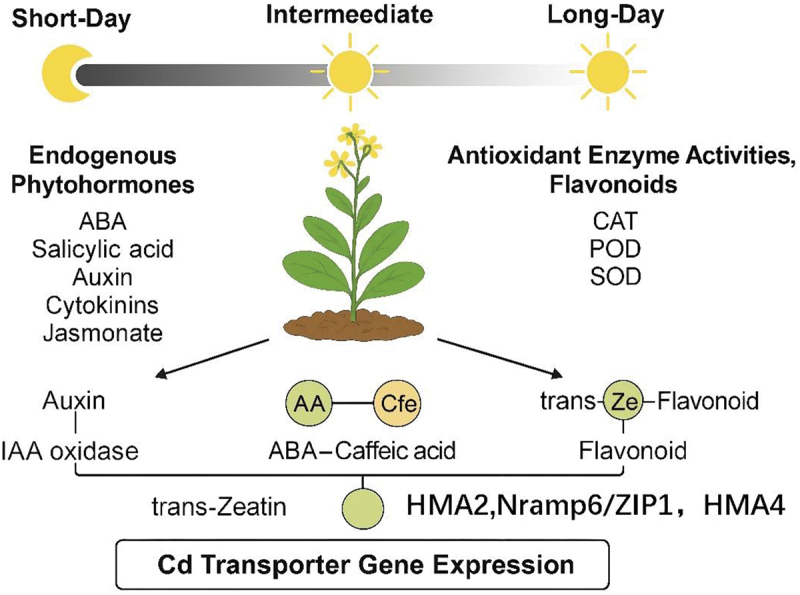
Molecular mechanisms of photoperiodic regulation in *Sedum alfredii*.

Photoperiod markedly reshapes *Sedum alfredii’*s hormonal and defense landscapes, with auxin, ABA, cytokinins, and flavonoids peaking alongside maximal antioxidant enzyme activities (CAT, POD, SOD) and Cd-transporter-related genes (*ZIP1*, *HMA4*, *NRAMP6*, *MT2*) expression under a 10-h photoperiod. CCA identified three core regulatory modules – auxin – IAAHyd, ABA – caffeic acid–*HMA4*/*ZIP1*, and trans-zeatin – flavonoid–*HMA2*/*NRAMP6*—positioning this day length as optimal for coordinating hormonal, metabolic, and genetic networks governing Cd uptake and tolerance. These insights provide a basis for designing photoperiod-based cultivation strategies to enhance *S. alfredii*‘s phytoremediation efficiency in Cd-contaminated soils (Figure 5).

## Ethical statement

All authors affirm that the research presented in this manuscript was conducted following the ethical standards of Molecular Biotechnology. The work presented did not involve any human or animal participants, nor did it include any biosafety experiments. The work has not been submitted elsewhere as a whole or in parts.

## Supplementary Material

Supplementary Material.docx

## Data Availability

All data generated or analyzed during this study will be available upon reasonable request.
