# Supplementary material for: Influence of photoperiod on endogenous phytohormone levels and cadmium-related gene expression in Sedum alfredii
Source: Plant Signal Behav. 2025 Aug 12;20(1):2544317. doi: 10.1080/15592324.2025.2544317 (PMC12344810; doi:10.1080/15592324.2025.2544317)
Supplement: Supplementary Material.docx [file KPSB_A_2544317_SM1590.docx]

Table S1. Photosynthetic photon flux density (PPFD) provided by the LED lamps used in this study.

| Treatment | PPFD |
| --- | --- |
| 2h | 161.99±23.53a |
| 4h | 170.51±8.75a |
| 6h | 168.73±9.59a |
| 8h | 163.64±20.44a |
| 10h | 173.66±14.84a |
| 12h | 169.55±29.77a |
| 14h | 172.17±18.96a |
| 16h | 172.48±12.71a |
| 18h | 165.08±7.20a |
| 20h | 174.24±20.93a |
| 22h | 173.23±6.00a |
| 24h | 170.66±26.46a |


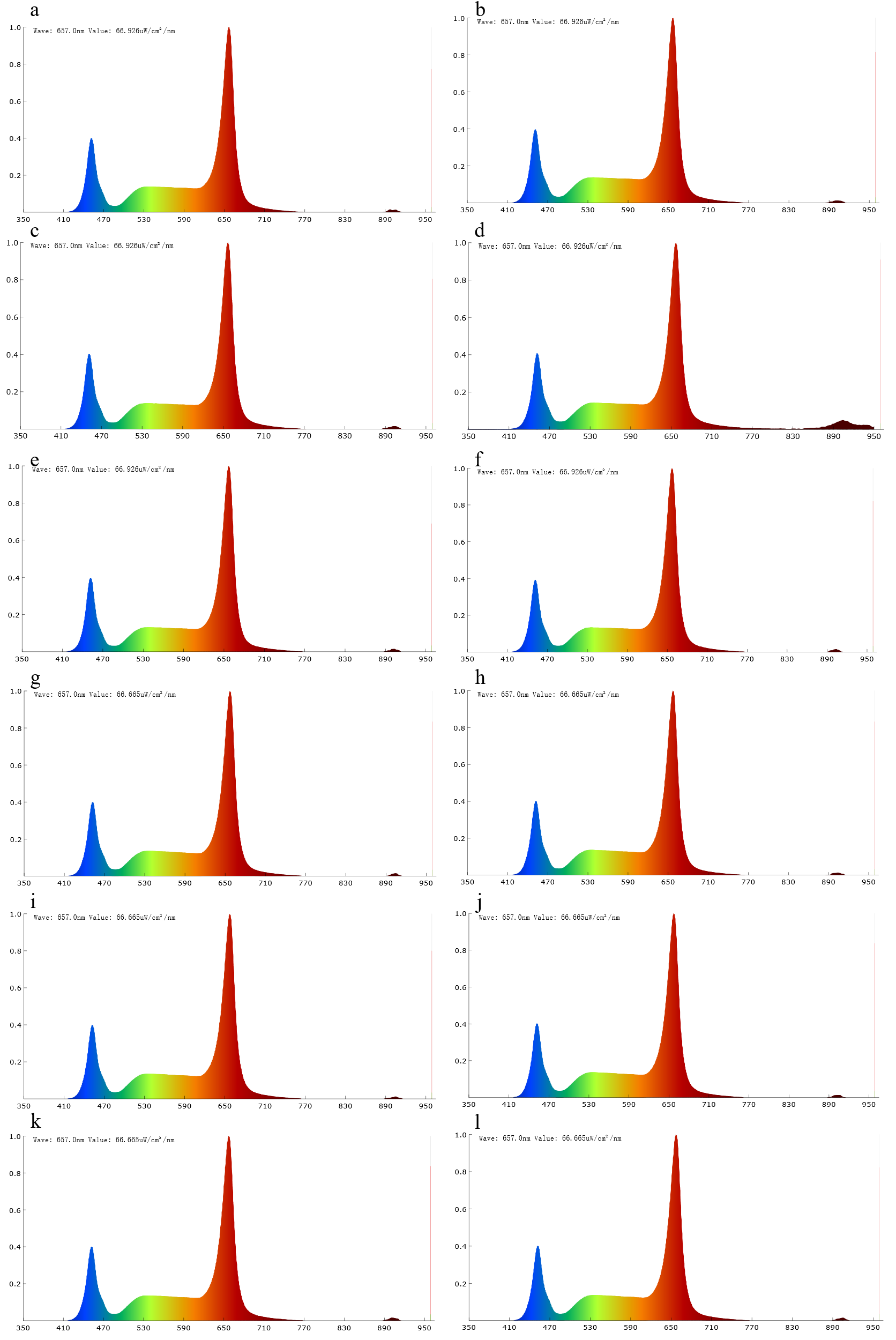


Fig. S1. Spectral distribution of the LED lamps used in this study, which were applied under photoperiod treatments of 2 (a), 4 (b), 6 (c), 8 (d), 10 (e), 12 (f), 14 (g), 16 (h), 18 (i), 20 (j), 22 (k), and 24 (l) hours per 24-hour cycle.

Table S2. Primer sequences of genes used for this study

| Genes | Forward primer | Reverse primer |
| --- | --- | --- |
| SaACTIN | TGTGCTTTCCCTCTATGCC | CGCTCAGCAGTGGTTGTG |
| SaNramp1 | CGCTGCTGTAGAGGATAAAGAC | CGGGATCAAGATATGCTAGGGA |
| SaNramp3 | AAGAAGCAGCTCATGGGTGT | TAAGCTGCGGTGAAGGTTGA |
| SaNramp5 | GTTGCTCTCATCGGTGGCTCAG | TCGCTGTGGAATTGGCATGTGA |
| SaNramp6 | TGTTTGGCGATTGTGCCAAG | ACATGCCAATTCCACAGCGA |
| SaZIP1 | AGGTGTTATCGGTATCTGCT | ACTTATCCCACGGAGTTTCA |
| SaZIP2 | GATTTGACGGAGAAGGAGTA | GTTTGAAGCGTTGCCTGATG |
| SaZIP3 | TCCCACTCGGTCATTATCGG | TTGGTGTTGTGATGGCGAAA |
| SaMT2 | CTGTGGTTGCGGATCTGCTT | TCCATTCTCCGACACCATCT |
| SaIRT1 | TGCTCCTGCTTCCGTTCA | TGAACGGAAGCAGGAGCA |
| SaZEP | TGAGCAGGCGGAAGGAATTAGGA | CCTTACCAACACCAATCGCATCCA |
| SaNCED | CGTGGTACGGCAAGTCGTCTTC | CGCATTCGGATTGGTGGATCAGAA |
| SaAAO | CGGTCAACTCGTATTCGCTGTCAA | CCACCTTCGCCGCAACTGATT |
| Sa8OH | CAATATGGTTGCCGATCTCGATGGA | CAAACACAGCCAGGATACCGACTT |
| SaIAA | TTCGTCGCAGCCGTTACTAGAGA | TTCGCCACAAGAAGTTCAGCAAATG |
| SaIAAHyd | CCTCCTCCTAGTCACATTCCACCTA | CTTCCTCATCCATTCAACGGTCTCC |
| SaGA3 | GCAGATGATGAATGGTGGTCGGTTA | CCATTGGACAGTGCCATGAAGGT |
| SatZ | AGCGATGACGACCATACTCTACACT | TGGTGACTCTGACTCCTCGTTGTG |
| SaSOD | TGACCTTGATGCTCTTGAACCGTTT | TCCACATAATTCCGATGGTGCCTTC |
| SaCAT | CGGCAGTCAGTGAGTTCTTGTTGT | TGCGGCATCGGTCGTTCATTC |
| SaPOD | TGTCTCGGTCTCCTCCAGATGC | GCCATTGTTGCGGTGCTGTAGA |
